# Standard versus extended pneumonectomy for lung cancer: what really matters?

**DOI:** 10.1186/1477-7819-12-248

**Published:** 2014-08-03

**Authors:** Dragan Subotic, Milan Savic, Nikola Atanasijadis, Milan Gajic, Jelena Stojsic, Marko Popovic, Vladimir Milenkovic, Zeljko Garabinovic

**Affiliations:** 1 Clinic for Thoracic Surgery, Clinical Center of Serbia, University of Belgrade School of Medicine, Koste Todorovica 26, 11000 Belgrade, Serbia; 2 Institute for Medical Statistics, University of Belgrade School of Medicine, Belgrade, Serbia; 3 Institute for Pathology, Clinical Center of Serbia, Belgrade, Serbia

## Abstract

**Background:**

It is still not clear whether an intrapericardial pneumonectomy indicates a more advanced stage of the disease compared to a standard pneumonectomy.

**Methods:**

This was a retrospective study of 164 patients who underwent a pneumonectomy for lung cancer. The first group consisted of 82 patients who had a standard pneumonectomy and the second group was 38 patients who had a intrapericardial pneumonectomy, for both groups in the latest 5-year period. The third group was 44 patients with had a sleeve pneumonectomy in the latest 10-year period. The groups were compared in relation to the overall and stage-related survival, influence of T and N factors, operative morbidity and mortality. The statistics used were Kaplan–Meier, U-test, *t*-test, χ^2^ test.

**Results:**

There was no statistically significant difference in stage distribution between standard and intrapericardial pneumonectomies; stages I, II, IIIA and IIIB occurred for 10.9% vs 2.6%, 30.5% vs 26.3%, 46.4% vs 65.8% and 12.2% vs 5.3% of patients, respectively. For patients who had a sleeve pneumonectomy, stage IIIA was significantly more frequent. Although the overall survival (63.5% vs 57.6%) and stage-related 5-year survival were better in the first compared to the second group, especially for stage IIIA (58.6% vs 42.6%), these differences were not statistically significant. There were no significant differences in operative morbidity and mortality between groups 1 and 2, but both were significantly higher in the third group (35.7% and 15.9%).

**Conclusions:**

An intrapericardial pneumonectomy does not always indicate a more advanced stage of the disease. The need for an intrapericardial pneumonectomy, either established preoperatively or during the operation, as a single factor, even for marginal surgical candidates, is not strong enough to reject these patients for surgery.

## Background

Despite a significant number of studies dealing with different aspects of pneumonectomy, some of the conclusions still remain unclear. The wide range for the reported major complication rate of 17 to 47% is not fully counterbalanced by the clearer situation for operative mortality, where a broad consensus exists that a rate of 8% should not be exceeded [1]. This is because standard and extended pneumonectomies are different operations and because intrapericardial pneumonectomy, without extension to neighbouring structures, is rarely, if at all, addressed in the literature.

An intrapericardial pneumonectomy may be necessary either because of the invasion by the tumour of the extrapericardial part of the pulmonary artery and/or vein, or due to the impossibility of safely dealing with the extrapericardial non-invaded part of the vessel as well. Finally, pericardial involvement by the tumour also requires this type of operation. Having in mind different reasons for the opening and partial resection of the pericardium, we set out to determine whether and in which way intrapericardial pneumonectomies, without an extension of the resection to adjacent structures, differ from standard pneumonectomies and sleeve pneumonectomies, which are a variant of true extended pneumonectomies.

An additional argument in favour of this study design is the conflicting data on the outcome of pneumonectomies in the era of neoadjuvant treatment. Usually with standard and extended pneumonectomies analysed together, there is a wide range in operative mortality, from 23.9% for the 90-day mortality rate after right pneumonectomy in the well-known report of Martini in 2001 [2], to 3.7% mortality in 27 patients undergoing pneumonectomy after induction therapy in a study by Perrot and colleagues [3].

## Methods

This was a retrospective study of 164 patients who had a pneumonectomy for primary lung cancer. The first group consisted of 82 patients who had a standard pneumonectomy, i.e. an extrapericardial pneumonectomy with systematic lymphadenectomy. The second group included 38 patients who had an intrapericardial pneumonectomy without resection of adjacent structures. Patients in these groups were operated on in the latest 5-year period. The third group consisted of 44 patients who had a sleeve pneumonectomy in the latest 10-year period.

In the present study, a standard pneumonectomy is defined as a pneumonectomy without opening the pericardium, with a routinely performed systematic lymphadenectomy. The term intrapericardial pneumonectomy in this study relates to a pneumonectomy without resection of the chest wall, left atrium, vena cava, tracheal bifurcation or oesophagus, but with opening of the pericardium (with or without its partial excision), because of the direct invasion of the tumour into the extrapericardial part of the pulmonary artery and/or vein (with or without pericardial involvement). A sleeve pneumonectomy refers only to a full-circumference anastomosis between the trachea and right or left (three patients) main bronchus.

### Inclusion criteria

Patients were included if they had undergone a complete resection (R0) and there was precise data about the pre-treatment tumour and patient characteristics and comorbidities; a detailed post-surgery pathohistological report; and precise data about postoperative complications, neoadjuvant or adjuvant therapy and treatment outcome.

### Preoperative work up

The preoperative work up for the assessment of the local extent of the lung cancer was the same in all groups (standard clinical and laboratory investigations, bronchoscopy, high-resolution computed tomography scan of the thorax and upper abdomen and respiratory function tests). For the staging of the mediastinum, a positron emission tomography (PET) scan was performed only for patients with a shorter lymph node diameter >1.5 cm. A mediastinoscopy was performed only for PET-positive patients.

For patients with moderate and severe chronic obstructive pulmonary disease (COPD), according to the GOLD criteria, we calculated the predicted postoperative forced expiratory volume in 1 second (ppoFEV1) using a perfusion lung scintigraphy with quantification of perfusion for each lung. A value of ppoFEV1 > 30% is accepted as a lower limit for safe lung resection. For all patients with cardiac comorbidity, a peak oxygen consumption of 15 ml.kg^-1^.min^-1^ served as a cut-off value for safe resection, according to current guidelines. The tumours were classified and staged according to the 2009 revision of the International System for Staging of Lung Cancer.

For all patients, bronchial closure was performed with a running suture (using polydioxanone (PDS) 2–0 cartilage to cartilage, membranous to membranous) with two or three reinforcing interrupted stitches (PDS 3–0). For patients undergoing a right pneumonectomy after neoadjuvant treatment, the bronchial suture line was almost routinely protected by the intercostal muscle flap, pericardial fat pad or diaphragm muscle flap.

### Data analysis

The demographic and clinical data were collected from the patients' original dossiers and at the time of outpatient visits, or by contacting the patients, their relatives or physicians by phone. All the data obtained were entered into the database. The groups were compared in relation to the overall and stage-related survival, influence of T and N factors, neoadjuvant treatment, operative morbidity and mortality. Survival time was calculated from the date of operation.

### Statistics

Survival was calculated using the Kaplan–Meier method. The log-rank test was used to compare survival between the groups; *P* < 0.05 was considered to indicate a statistically significant result. All statistical analyses were performed using SPSS software (version 13.0 for Windows). A multivariate analysis was used to evaluate the effect of different covariates on the treatment outcome and complication rate. Other statistical tests used were the U-test to analyse disease stage distribution throughout the analysed groups; the *t* test to compare ventilatory parameters and the χ^2^ test for an intergroup comparison of operative morbidity, mortality and bronchopleural fistula occurrence. The study was approved by the institutional review board of the Clinic for thoracic surgery of the Clinical center of Serbia in Belgrade.

## Results

### Group structure, baseline lung function and disease stage distribution

The structure of the analysed groups is presented in Table 1. There were no significant differences in the age of the patients; the mean ages for the standard, intrapericardial and sleeve pneumonectomy groups were 58.9 ± 7.8, 58.7 ± 6.2 and 51.4 ± 5.7 years, respectively.

**Table 1:** Structure of the analysed groups

		**Group 1**		**Group 2**		**Group 3**		***P***
Age (mean ± standard deviation) (years)		58.9 ± 7.8		58.7 ± 6.2		51.4 ± 5.7		<0.05
Side (right/left)		38/44		22/16		41/3		G1:G2: *P* > 0.05
								G1 + G2 vs G3: *P* < 0.05
		*n* (%)		*n* (%)		*n* (%)		
Operative stage	Ia	2	(2.4)	1	(2.6)	0	(0)	>0.05
	Ib	7	(8.5)	0	(0)	0	(0)	
	IIa	17	(20.7)	3	(7.9)	0	(0)	
	IIb	8	(9.8)	7	(18.4)	0	(0)	
	IIIa	38	(46.4)	25	(65.8)	26	(59)	
	IIIb	10	(12.2)	2	(5.3)	18	(41)	
COPD	Mild	10	(12.2)	4	(10.5)	5	(11.3)	COPD total
	Moderate	9	(10.9)	4	(10.5)	9	(20.5)	G1: G2:G3
	Severe	0	(0)	0	(0)	1	(2.2)	*P* = 0.196
	COPD total	19	(23.1)	8	(21)	15	(34)	
Cardiovascular risk		10	(12.2)	5	(13.2)	6	(13.6)	>0.05
Other comorbidities*		10	(12.2)	5	(13.2)	5	(11.4)	>0.05
Neoadjuvant therapy		22	(26.8)	12	(31.6)	5	(11.4)	G1:G2: *P* > 0.05
								G1 + G2 vs G3: *P* < 0.05
Adjuvant therapy		30	(36.5)	15	(39.4)	19	(43.2)	>0.05
Tumour histology	Squamous	55	(67)	26	(68.4)	35	(79.6)	>0.05
	Adeno Ca	24	(29.2)	10	(26.3)	5	(11.4)	
	Other	3	(3.6)	2	(5.3)	4	(9.1)	
T component	T1	9	(10.9)	4	(10.5)	0	(0)	G1 + G2 vs G3
	T2	37	(45.1)	10	(26.3)	0	(0)	<0.05
	T3	21	(25.6)	19	(50)	16	(36.4)	
	T4	15	(18.3)	5	(13.2)	18	(32.14)	
N2 lesions		25	(30.5)	12	(31.6)	22	(50)	<0.05

G1, group 1; G2, group 2; G3, group 3.

For patients who had a standard or intrapericardial pneumonectomy, the proportion of right- vs left-sided tumours was 38 vs 44 and 22 vs 16, respectively. For patients who had a sleeve pneumonectomy, the right-sided operation was performed for 41 patients, whilst for 3 patients a one-stage left sleeve pneumonectomy was done.

The preoperative lung function was well preserved in all groups. There was no significant difference in the baseline lung function parameters between the analysed groups. In the standard pneumonectomy group, COPD was diagnosed for 19 (23.17%) patients. In the intrapericardial pneumonectomy and sleeve pneumonectomy group, there were 8 (21%) and 15 (34%) patients with COPD, respectively.

Despite a clearly higher proportion of postoperative stages IIB and IIIA in the intrapericardial group vs standard pneumonectomy group, there was no statistically significant difference in stage distribution between the first and second groups (standard vs intrapericardial pneumonectomy), where stages I, II, IIIA and IIIB were for 10.9% vs 2.6%, 30.5% vs 26.3%, 46.4% vs 65.8% and 12.2% vs 5.3% respectively.

Ten patients who had a standard pneumonectomy were classified as stage IIIB based on the involvement of the oesophageal muscular layer (two patients), N3 lesions (one patient), additional lung nodules in the non-primary tumour lobes (three patients), direct phrenic nerve involvement (two patients) and mediastinal fat pad involvement (two patients). For patients who had a sleeve pneumonectomy, stage IIIA was for 26 patients (59%) and stage IIIB for 18 patients (41%).

### Comorbidity, adjuvant/neoadjuvant treatment, operative morbidity and mortality

For the preoperative comorbidities, for patients who had a standard or intrapericardial pneumonectomy, COPD was diagnosed for 19 (23.17%) and 8 (21%) patients, respectively. A major cardiovascular comorbidity was found for 10 (12.2%) and 5 (13.2%) patients, respectively. These differences were not statistically significant.

In the sleeve-pneumonectomy group, major cardiovascular risk factors were identified in 6 patients (13.6%) (two of whom died in the early postoperative period), whilst COPD was found in 15 patients (34%). The evidently higher proportion of COPD patients in the latter group, compared with the first two groups, was not significantly significant.

Other comorbidities, like diabetes, gastric ulcer, previous CVI and chronic renal failure, were found for the first, second and third groups for 10 (12.2%), 5 (13.2%) and 5 (11.365%) patients, respectively. There were no significant differences between the analysed groups for these comorbidities.

In the standard pneumonectomy group, postoperative complications occurred in 7/39 (17.95%) patients with a comorbidity and in 4/43 (9.35) patients without a comorbidity. In the intrapericardial pneumonectomy group, postoperative complications in patients with and without a comorbidity were registered in 4/18 (22.2%) and 3/20 (15%) patients, respectively. Although the postoperative complications were more frequent in patients with a comorbidity, these differences were not statistically significant, neither in these two, nor in the sleeve pneumonectomy group.

Postoperative complications occurred more frequently in patients with COPD (22%) and cardiovascular diseases (20%) compared to other types of comorbidity (13.3%), but these differences were not statistically significant.

Preoperative chemotherapy was administered to 22 patients (26.8%) who had a standard pneumonectomy, to 12 patients (31.5%) who had an intrapericardial pneumonectomy and to 5 (11.3%) patients who had a sleeve pneumonectomy.

In the first and second groups, postoperative adjuvant chemo/radiation therapy was administered to 30 (36.5%) and 15 (39.4%) patients, respectively. In the sleeve pneumonectomy group, postoperative adjuvant treatment was given to 19 patients (43.2%). Seven patients received chemotherapy and 12 were irradiated postoperatively. For only one patient, who was irradiated postoperatively, pleural empyema without a bronchopleural fistula occurred.

In the first, second and third groups, postoperative complications within 30 postoperative days occurred in 11/82 (13.4%), 7/38 (18.4%) and 15/44 (34%) respectively (Table 2). Operative morbidity was significantly higher in the sleeve pneumonectomy compared with other two groups. Although bronchopleural fistulas occurred more frequently in the sleeve pneumonectomy group (6/44, 13.6%) compared to the first (5, 6.09%) and second (1, 2.6%) groups, these differences were not statistically significant.

**Table 2:** Postoperative morbidity, mortality and bronchopleural fistula occurrence

	**Group 1**		**Group 2**		**Group 3**		**χ**^**2**^	***P***
	***n *** **(%)**		***n *** **(%)**		***n *** **(%)**			
Postoperative morbidity	11/82	(13.4)	7/38	(18.4)	15/44	(34)	7,705	0.021
Postoperative mortality	0	(0)	0	(0)	7/44	(15.9)	*	**
Bronchopleural fistula	5/82	(6.1)	1/38	(2.6)	6/44	(13.6)	4,001	0.135

*Group 1 vs group 3 (χ ^2^ = 10.947); group 2 vs group 3 (χ ^2^ = 4.729).

**Group 1 vs group 3 (*P* = 0.029); group 2 vs group 3 (*P* < 0.001).

In the sleeve-pneumonectomy group, the postoperative complications were: bronchopleural fistula (six patients), pulmonary embolism (one), myocardial infarction (one), gastric stress ulcer (one), pleural empyema (two) and cardiac arrhythmias (four).

Among several analysed factors, only age and postoperative T factor were found to be significantly unfavourable in terms of the bronchopleural fistula occurrence for the first and second groups (Table 3). Age, sex, tumour side, preoperative lung function, neoadjuvant treatment and postoperative N factor were not confirmed as significant contributive factors. In the sleeve-pneumonectomy group, T (hazard ratio 2.37, *P* < 0.04) and N component (hazard ratio 2.99, *P* < 0.01) were found to be significant.

**Table 3:** Factors influencing the occurrence of a bronchopleural fistula

**Factors**	***B***	**SE**	**Df**	**Sig.**	**Exp ( *B *)**	**95% confidence interval lower/upper**
Sex	−0.795	0.857	1	0.354	0.452	0.084/2.423
Age	0.135	0.061	1	**0.026**	1.145	1.016/1.291
FEV1 (%)	0.018	0.026	1	0.490	1.018	0.968/1.070
Tumour side	0.014	0.597	1	0.982	1.014	0.314/3.268
pT*	1.390	0.424	1	**0.001**	4.014	1.749/9.213
pN**	0.796	0.576	1	0.167	2.217	0.717/6.861
Neoadjuvant	−0.579	0.800	1	0.469	0.561	0.117/2.687

*Significant in all groups.

**Significant only for the third group (hazard ratio 2.99, *P* < 0.01).

In the first two groups, no patients died within the first 30 postoperative days. In the sleeve-pneumonectomy group, operative mortality was 7/44 (15.95%). Of the seven patients who died, four had a bronchopleural fistula, whilst a pulmonary embolism, myocardial infarction and gastric ulcer occurred in one patient each.

### Survival

**Figure 1:**
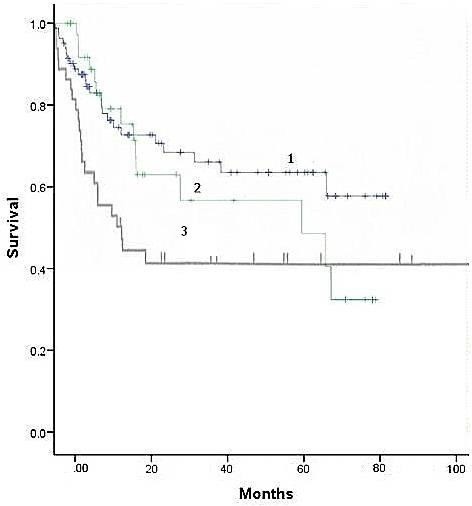
**Overall survival.** 1: Standard pneumonectomy. 2: Intrapericardial pneumonectomy. 3: Sleeve pneumonectomy

**Figure 2:**
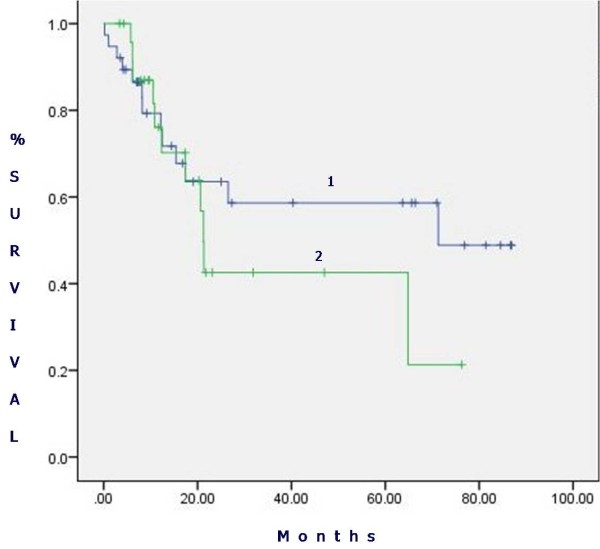
**Survival for stage IIIA patients (groups 1 and 2).** 1: Standard pneumonectomy. 2: Intrapericardial pneumonectomy.

**Figure 3:**
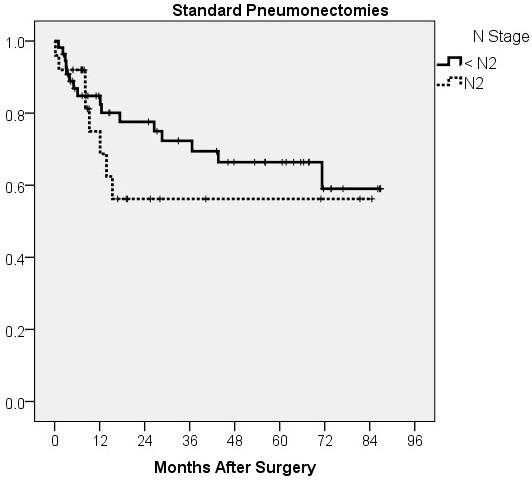
Survival depending on nodal stage for patients who had a standard pneumonectomy.

**Figure 4:**
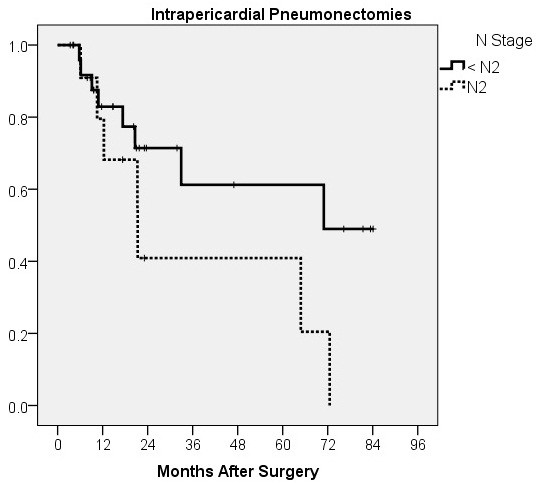
Survival depending on nodal stage for patients who had a intrapericardial pneumonectomy.

**Figure 5:**
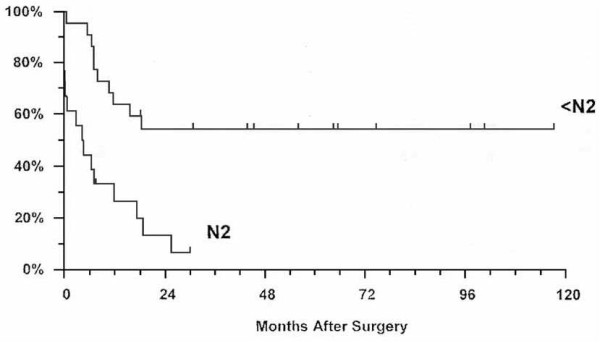
Survival depending on nodal stage for patients who had a sleeve pneumonectomy.

The survival of patients in the analysed groups is presented in Figure 1. The 5-year survival of patients who had a standard, intrapericardial or sleeve pneumonectomy was 63.5%, 57.6% and 35%, respectively. The mean survival of patients who had a standard or intrapericardial pneumonectomy was 59.6 ± 4.5 and 51.3 ± 6.2 months, respectively. Despite evident survival differences for stage IIIA patients (Figure 2), there was no significant difference in stage-related survival between groups 1 and 2 (Table 4). Survival depending on nodal stage in patients who had a standard or intrapericardial pneumonectomy is presented in Figures 3 and 4, respectively. For patients who had a sleeve pneumonectomy, there was a significant survival difference between patients with N0 + N1 vs N2 lesions (Figure 5).

**Table 4:** Significance of stage-related survival for groups 1 and 2

**Operative stage**	**Standard (group 1)**		**Intrapericardial (group 2)**		**DF**	**Sig.**
	**Mean**	**Standard deviation**	**Mean**	**Standard deviation**	**Mean**	**Standard deviation**
IIB	74.5	10.7	54.2	8.3	1	0.273
IIIA	54.0	7.1	38.3	7.2	1	0.399
IIIB	18.1	4.1	72.5	0	1	0.123

The tumour side did not significantly influence survival in the analysed groups (Table 5). There were only three left-sided operations in the sleeve-pneumonectomy group (one patient died on postoperative day 5 from a massive pulmonary embolism, another survived one year), which does not allow reliable conclusions to be reached.

**Table 5:** Survival in groups 1 and 2 depending on tumour side

**Procedure**	**Tumour side**	**Mean**	**Standard error**	**Lower**	**Upper**	**Chi squared**	**Df**	**Sig.**
Standard pneumonectomy	Right	57.82	6.45	44.63	69.93			
	Left	55.08	4.94	45.40	64.76	0.106	1	0.745
Intrapericardial pneumonectomy	Right	55.60	8.11	39.69	71.50			
	Left	51.28	6.23	39.06	63.49	0.667	1	0.414

## Discussion

### Stage distribution

In the present study, the term standard pneumonectomy refers only to pneumonectomy with systematic lymphadenectomy, but without opening the pericardium and without resection of other structures. In some studies, however, standard pneumonectomy was defined in the same way, but including both intrapericardial and extrapericardial lung removal [4]. The reason for our definition was that for the study end point we aimed to assess eventual differences between the two types of operation.

It should be mentioned that the proportion of pneumonectomies at early disease stages may be quite high, reaching nearly 30% in some series, like in a recent multicentre review of 1,475 patients with pathologic stage I or II NSCLC, where 421 (28.5%) patients underwent a pneumonectomy and 1,054 (71.5%) underwent a lobectomy or bilobectomy [5]. Moreover, after adjusting for age, primary tumour status, regional nodal status and preoperative FEV1, it was also shown that survival after pneumonectomy may not differ significantly compared with lobectomy/bilobectomy [6].

Concerning the stage distribution, an adequate comparison is possible with the small number of studies specifically addressing an intrapericardial operation as a single factor, like in a series of 130 intrapericardial pneumonectomies [7]. Our finding of the absence of significant differences for the stage distribution between the standard and intrapericardial pneumonectomy groups is caused by the relatively high proportion of lower disease stages in the intrapericardial group – 26.3%, which is similar to the aforementioned study with 30/130 (23%) patients in stage II. The relatively high proportion of T2 tumours in that study – 70/130 (53.8%), 2 with N0, 28 with N1 and 40 with N2 – confirms that the decision for the opening of the pericardium does not always reflect a more advanced local spread, but also tumour location. In addition, the proportion of stage IIIA patients in that study 86/130 (66%) was somewhere in between the proportion of that stage in our standard (58.5%) and intrapericardial pneumonectomy groups (71.1%).

In brief, a pneumonectomy in early disease stages may have two opposed effects: an unfavourable effect (especially for a right pneumonectomy) on cardiorespiratory function and a more favourable one through the reduced risk of local relapse, possibly due to a better clearance of N1 lymph nodes, as suggested by Luzzi and colleagues [8].

### Operative morbidity and mortality

The almost identical major cardiovascular comorbidity rate (12% to 13%) and similar proportion of COPD patients in the present study, minimized the possible influence of comorbidity on operative morbidity and mortality. The absence of 30-day mortality and similar morbidity rates (13.4% and 18.45%) in the first two groups of the present study are on the lower end of the reported 12% to 37% complication rate, even without neoadjuvant therapy. The rare reports of mortality and morbidity exclusively after intrapericardial pneumonectomy have rates ranging from 5% to 10% and reaching 20% [9,10].

The significantly higher morbidity (34%) and mortality (16%) rates in the sleeve-pneumonectomy group, mirrored the experience of Tsuchiya and Watanabe, who reported an operative mortality of 15% to 17% as an acceptable risk [11]. In the sleeve-pneumonectomy group, the cardiovascular complication rate was slightly lower than the rate reported in the literature, and is the same as for standard pneumonectomy [12].

One of the possible causes of the high mortality and morbidity rate in the sleeve-pneumonectomy group could be the unexpected sleeve resection. In the present study, a sleeve resection was not expected before the operation for 11 patients (25%). Except for the study of Porhanov *et al*. [13], which is the world’s largest experience of sleeve pneumonectomy with 189 patients operated on, other papers do not address this problem. In that study, the necessity for a sleeve pneumonectomy was revealed during the operation for 23% of patients, but the causes were not analysed. In our group, all such patients had either a tumour in the main bronchus or in the upper lobe, but always with invasion of the main carina from outside the bronchial wall.

The reason for focusing on factors influencing the occurrence of a bronchopleural fistula in the present study, was our observation that cardiac disorders or respiratory insufficiency, usually reported as separate complications, may be direct consequences or even the first sign of a bronchopleural fistula. We can only speculate that the quite low bronchopleural fistula rate in the present study (6.09% and 2.6% for standard and intrapericardial pneumonectomies) can be explained by our technique of manual closure of the bronchus (as described); this technique was used for all patients.

In contrast to the literature data, which shows that a right-sided pneumonectomy is associated with a mortality of 12% to 37% even in the absence of neoadjuvant chemoradiation therapy, in the present study the tumour side was not significant in terms of the occurrence of a bronchopleural fistula. Although this finding is similar to those of the small number of studies that have found no significant influence for the tumour side [14,15], these studies were smaller, with data collected over many years. In studies with a high reported operative mortality (20% 90-day mortality after right-sided vs 9% after left-sided pneumonectomy, *P* = 0.084), there was a high rate (12%) of bronchopleural fistula as well [16]. Furthermore, in some studies the significant difference in major operative morbidity depending on tumour side (with a right vs left respiratory failure rate of 37% vs 8%), was associated with significant survival differences in favour of left-sided tumours [4].

Unlike the tumour side, patients’ age was found to be significant in terms of the occurrence of a bronchopleural fistula, which is in line with most literature data, as only a few studies support the lack of an association between advanced age and morbidity [17]. There is still some controversy related to the age limit for a safe resection. According to UKPOS, patients older than 62 years had up to a fivefold increase in the rate of major complications. The American College of Cardiology and American Heart Association (ACC/AHA) practice guideline indicates that patients of 70 years or older carry a particularly high risk for perioperative cardiac morbidity [18]. The median age of our study population of 59 years is similar to that reported in publications on patients undergoing a pneumonectomy for lung cancer, which is 58 to 65 years [19]. As the number of patients older than 65 years undergoing thoracic surgery is estimated to increase from the current 6 million to nearly 12 million per year, we do not limit surgery to the proven low-risk age group; in the present study, 8 patients were older than 70 years in the first group and one in the second group.

The absence of the significant influence of FEV_1_ on operative morbidity is probably a consequence of the quite low number of COPD patients – 23.17% in the first and 21% in the second group. We also avoided sacrificing the phrenic nerve whenever possible during the intrapericardial pneumonectomies, by mobilising and retracting it medially. This is because it has been clearly demonstrated that preserving the phrenic nerve may be beneficial in terms of postoperative lung function [20]. Although a significantly higher rate of bronchopleural fistula in the sleeve-pneumonectomy group coincided with a quite high (36.36%) proportion of COPD patients, this complication cannot be attributed to COPD, as for the 4/6 patients who died with a bronchopleural fistula, the length of the resected tracheo-bronchial segment was >4 cm. The literature data are conflicting: only a few studies have found FEV_1_ to contribute as a predictor of morbidity or mortality in multivariate analysis [21] and also a number of studies have not confirmed such an association [15,16]. That is why in the present study, like in our previous experience with COPD patients, beside the FEV_1,_ we included in the analysis the small airways function (FEF_50_, FEF_25_) as well. Although we previously demonstrated that this part of the lung function may be significantly improved preoperatively [22], no significant influence on operative morbidity was demonstrated in the present study.

Due to the retrospective nature of the study, in the analysis of the influence of neoadjuvant treatment (mostly chemotherapy), the inconsistent policy for N2 lesions should be kept in mind. This relates more to the first two groups, in which 26.8% and 31.5% patients received neoadjuvant treatment, than for the sleeve-pneumonectomy group with 11.3% such patients. Our results support the results of studies that have recently challenged the traditionally reported high complication rate after this type of treatment; there was 3.7% mortality in the study by Perrot and colleagues [3] and 6.7% mortality after neoadjuvant vs 5.5% for 238 patients undergoing surgical treatment alone in the series described by Mansour and colleagues [23]. That there were no deaths within the first 30 postoperative days in the first two groups of the present study, may be attributed to the absence of preoperative irradiation therapy for these groups. In addition, for right-sided pneumonectomies, the bronchial suture line was almost routinely protected as previously described.

### Survival

The slightly superior survival rates in the first two analysed groups (63.55% and 57.6%) compared with those reported in many other series, was due to the higher proportion of lower disease stages in both groups and to the shorter follow-up of patients operated later in the latest 5-year period. On the other hand, the reported survival after neoadjuvant treatment (33 to 46%) for patients with a regionally advanced disease [24], confirms that the long-term results after pneumonectomy, even for these unfavourable groups, may be acceptable. However, one of the main study end points was the comparison of survival between the first and second groups. The absence of a significant difference in stage-related survival between groups 1 and 2, despite evident survival differences for stage IIIA patients, strongly supports our initial clinical observations that extension of the resection into the pericardium does not necessarily mean a worse outcome.

Survival after sleeve pneumonectomy reflects the major differences between the groups from the standpoint of patient selection, operative risk and final outcome. The 5-year survival of patients in our group is better than that found by several studies with 16 to 40 patients, but is still lower than the 43% survival rate reported by Dartevelle and Macchiarini [25]. The significant survival difference in favour of patients without pN_2_ lesions vs patients with pN_2_ lesions in our series clearly supports the attitude that this operation should not be performed for patients with N2 lesions that are confirmed either before or during thoracotomy. The literature data are heterogeneous regarding the significance of nodal stage; attitudes vary from not accepting suspected N2 lesions as a contraindication to surgery, through performing mediastinoscopy only in the presence of tracheal compression above the anticipated line of resection, to routine mediastinoscopy for all candidates for this operation [26].

### Study limitations

A comparison of patients who have had a standard, intrapericardial or sleeve pneumonectomy as separate groups might seem debatable. However, in the Methods section, the explanation for this approach was given and was subsequently confirmed by the results, which show that standard and intrapericardial pneumonectomies (without extension to surrounding structures) are not necessarily associated with more extensive disease spread, regardless of whether the pericardium was only opened or partially resected. An intrapericardial pneumonectomy is by no means more similar to a sleeve pneumonectomy than to a standard pneumonectomy. A sleeve pneumonectomy is a variant of an extended pneumonectomy and, as such, completely different from the other two operations.

## Conclusions

To conclude, the operative risk and outcome after an intrapericardial pneumonectomy, without resection of surrounding structures, are comparable to those after a standard pneumonectomy. If the need for an intrapericardial pneumonectomy is determined either preoperatively or during the operation, this fact, even for marginal surgical candidates, is not strong enough to reject these patients for surgery.

## Consent

Written informed consent was obtained from the patient for the publication of this report and any accompanying images.

## Abbreviations

COPD: Chronic obstructive pulmonary disease; FEV1: Forced expiratory volume in 1 second; PET: Positron emission tomography; ppoFEV1: Predicted postoperative forced expiratory volume in 1 second.

## Competing interests

The authors declare that they have no competing interests.

## Authors’ contributions

DS: study design, surgery, data analysis, manuscript draft; MS: surgery, data acquisition, data analysis, manuscript draft; NA: study design, surgery, manuscript draft; MG: study design, statistical analysis; JS: study design, pathological analysis of all operative specimens; MP: data acquisition and statistical analysis; VM: surgery, data acquisition; ZG: surgery, data acquisition and analysis. All authors read and approved the final manuscript.

## Authors’ information

This research was presented at the 27th EACTS Annual Meeting, 5–9 October 2013, in Vienna.
